# Supplementary material for: Circulating Human Serum Metabolites Derived from the Intake of a Saffron Extract (Safr’InsideTM) Protect Neurons from Oxidative Stress: Consideration for Depressive Disorders
Source: Nutrients. 2022 Apr 5;14(7):1511. doi: 10.3390/nu14071511 (PMC9002571; doi:10.3390/nu14071511)
Supplement: Supplementary file 1 [file nutrients-14-01511-s001.zip › nutrients-1658521-supplementary.pdf]

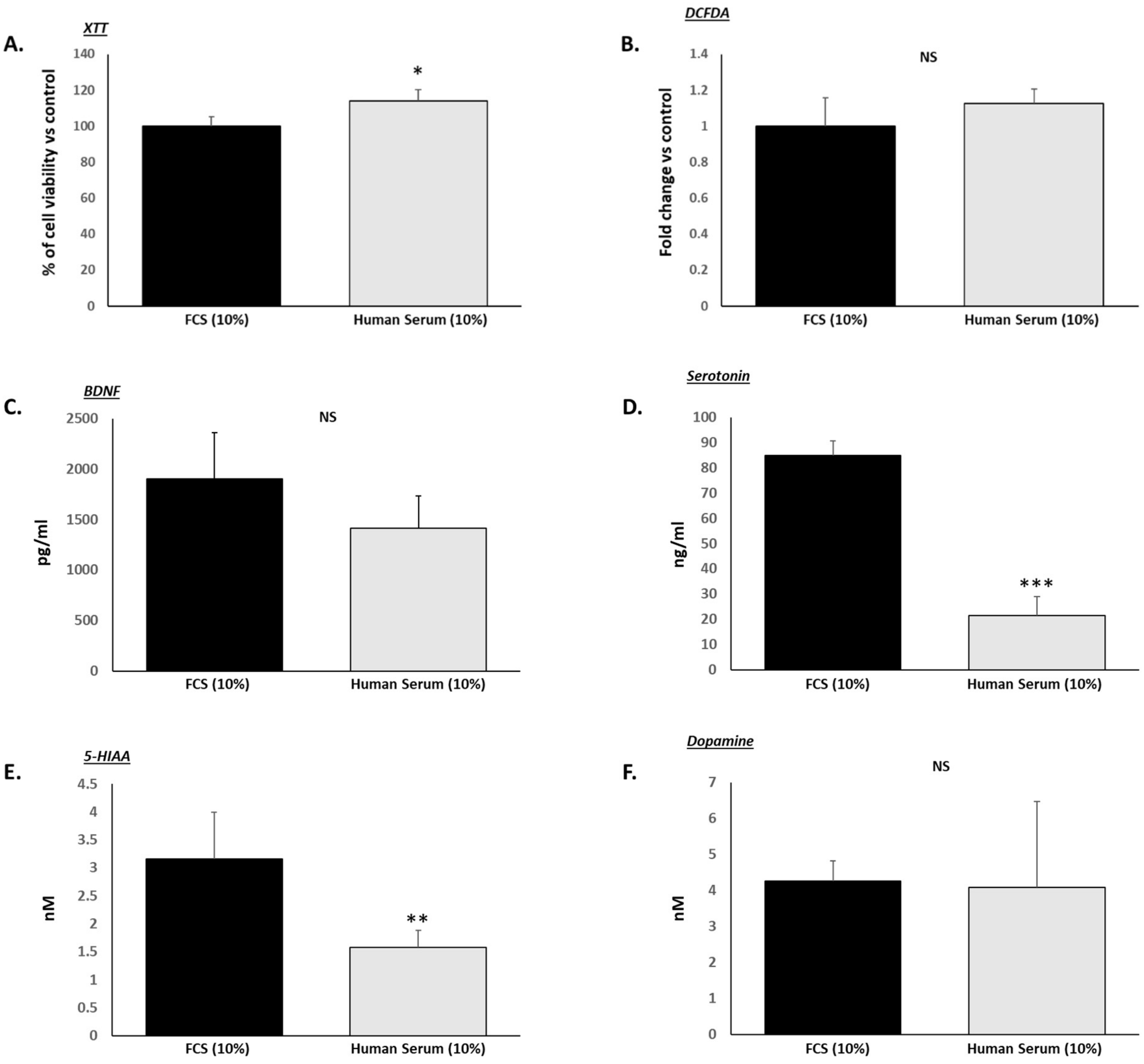

Figure S1 : Effect of human serum on viability, ROS levels, BDNF and select neurotransmitters in differentiated SH-SY5Y cells. Differentiated SH-SY5Y were incubated with serum from calf or human origin for 48h. Cellular viability was measured using a XTT-based assay (A) and intracellular ROS levels with the DCFDA probe (B). BDNF (C), Serotonin (D), 5-HIAA (E) and Dopamine (F) were all measured by ELISA. Human serum led to a reduction of both Serotonin and 5-HIAA levels. It had no major effect on other parameters. * ($P < 0.05$); ** ($P < 0.01$); *** ($P < 0.001$).

Along with oxidative stress impairment, the pathophysiology of depression includes glucocorticoids excess. Indeed, corticosterone is a common inducer of a depressive-like behavior in rodents. Interestingly, in such preclinical models, alteration of behavior is accompanied by the increase of oxidative stress markers (lipid peroxidation, nitrite, and protein carbonyl) and the decrease in nonprotein thiols level [1, 2]. Moreover, in a rat social defeat model of psychological stress, the use of nutrients with antioxidant properties (grape powder) reverses behavioral and cognitive alterations by preventing increase of both corticosterone and 8-isoprostane levels in serum [3]. Consistent with these data we found that corticosterone induced oxidative stress in human neurons supporting the relevance of a central oxidative mechanism for mimicking a depression context and validating further the use of H2O2 in our cell culture investigations.

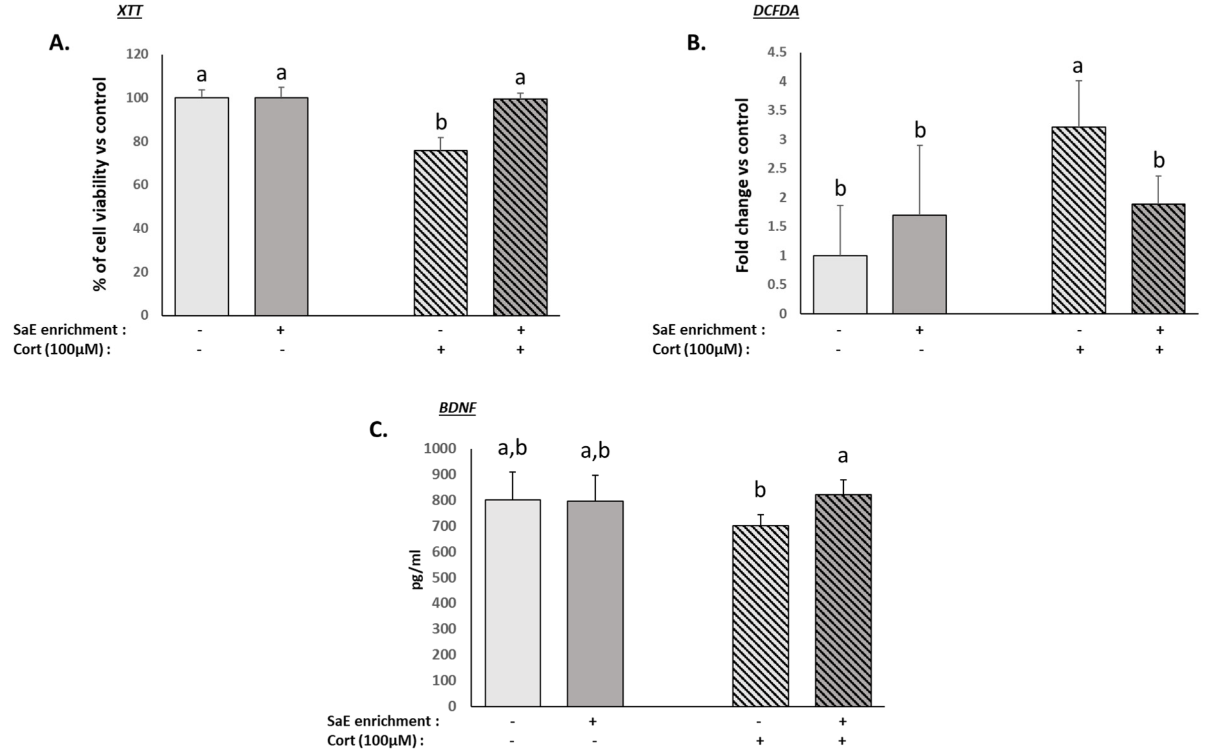

Figure S2 : Effect of human serum enriched with SaE metabolites on the cellular injuries caused by corticosterone in SH-SY5Y cells. Differentiated SH-SY5Y were incubated with serum from human origin for 24h prior to an additional 24h treatment with 100µM corticosterone (Sigma-Aldrich, C2505). Cellular viability was measured using a XTT-based assay (A). Intracellular ROS levels (B) as well as BDNF production (C) were measured using DCFDA and ELISA-based assays respectively. SaE enrichment was able to significantly counteract the decreased viability (A) and BDNF production (C) as well as the increase of intracellular ROS (B) caused by H2O2 treatment. Results are expressed as mean +/− SEM (standard error of the mean). Statistical analyses were carried out using ExcelStat Pro (Microsoft, Issy-les-Moulineaux, France). One-way ANOVA followed by Fisher post hoc test were performed. Groups significantly different from each other ($p < 0.05$) are indicated with different letters. Groups with no significant statistical difference from each other share the same letter. XTT: global ANOVA *p-value <0.001* and *F-value: 22.150*; DCFDA: global ANOVA *p-value =0.004* and *F-value: 4.784*; BDNF: global ANOVA *p-value =0.016* and *F-value: 3.670.*

1. Camargo, A., et al., *Cholecalciferol counteracts depressive-like behavior and oxidative stress induced by repeated corticosterone treatment in mice.* Eur J Pharmacol, 2018. **833**: p. 451-461.
2. Dalmagro, A.P., et al., *Morus nigra leaves extract revokes the depressive-like behavior, oxidative stress, and hippocampal damage induced by corticosterone: a pivotal role of the phenolic syringic acid.* Behav Pharmacol, 2020. **31**(4): p. 397-406.
3. Solanki, N., et al., *Modulating Oxidative Stress Relieves Stress-Induced Behavioral and Cognitive Impairments in Rats.* Int J Neuropsychopharmacol, 2017. **20**(7): p. 550-561.
